# Zoledronic acid regulates the synthesis and secretion of IL-1β through Histone methylation in macrophages

**DOI:** 10.1038/s41420-020-0273-4

**Published:** 2020-06-11

**Authors:** Xiaojie Yang, Xing Xu, Jun Chen, Qing Wang, Guangfei Wang, Xuemin Ai, Xu Wang, Jinsong Pan

**Affiliations:** 1 Department of Stomatology, Shanghai General Hospital, Shanghai Jiao Tong University School of Medicine, Shanghai, China; 2 Department of Oral and Maxillofacial-Head and Neck Oncology, Ninth People’s Hospital, Shanghai Jiao Tong University School of Medicine, Shanghai, China

**Keywords:** Epigenetics, Inflammation

## Abstract

Long-term administration of nitrogen-containing bisphosphonates increases the risk of detrimental side effects, such as bisphosphonate-related osteonecrosis of the jaw (BRONJ). BRONJ development is associated with inflammation, but its pathophysiology remains unknown. Here, we examined whether histone methylation is responsible for zoledronic acid (Zol)-induced inflammatory responses. We found that Kdm6a and Kdm6b markedly increased interleukin 1β expression and Gasdermin D cleavage, which are both activated by Caspase 1, in macrophages. Inhibitors of Kdm6a and Kdm6b robustly abolished Zol-enhanced interleukin 1β synthesis and secretion from macrophages. When Kdm6a and Kdm6b were pharmacologically inhibited in vivo, poor healing of the alveolar socket and inflammatory responses were ameliorated in Zol-treated mice. Taken together, we showed the pathologic role of Kdm6a and Kdm6b in Zol-promoted inflammatory responses and demonstrated that Kdm6a and Kdm6b are potential therapeutic targets for the treatment of BRONJ.

## Introduction

Bisphosphonates (BPs) are highly associated with bone minerals and exhibit potent anti-bone-resorptive effects. They are widely used for bone-metastatic cancers, multiple myeloma, osteoporosis, osteogenesis imperfecta and Paget’s disease^1^. Since reported by Marx in 2003^2^, bisphosphonate-related osteonecrosis of the jaw (BRONJ) has emerged as a serious side effect of BP treatment, especially nitrogen-containing bisphosphonates (NBPs), such as zoledronic acid (Zol)^3^. Risk factors such as operative treatment, concomitant oral disease, chemotherapeutics, anti-angiogenic agents and steroids are related to the pathogenesis of BRONJ^4^. Although significant improvements have been made with respect to risk reduction strategies, prevention and treatment^5^, there is still an inadequate understanding of the underlying mechanisms of BRONJ and a need for more effective therapeutic approaches.

Caspase 1 activation and IL-1β production in macrophages contribute to the development of BRONJ^1,4^. Macrophages play an important role in responding to inflammation to maintain tissue homoeostasis^6^. When macrophages are stimulated by pathogen-associated molecular patterns and damage-associated molecular patterns, protein complexes called inflammasomes are induced that recruit pro-Caspase 1 and result in its proximity-mediated auto-proteolysis. Then, active Caspase 1 matures the cytokines pro-IL-1β and pro-IL-18. The critical pyroptosis mediator Gasdermin D (GSDMD), which normally exists in the autoinhibited state, can also be cleaved by active Caspase 1 in a pathway that was initially called “canonical inflammasome” activation^7^. Cleavage of GSDMD relieves an intramolecular auto-inhibition and enables translocation of the N-terminal fragment of GSDMD to the inner leaflet of the plasma membrane to form pores. Thus, IL-1β and IL-18 are released extracellularly to stimulate inflammatory responses^8,9^. Pyroptosis has been suggested to contribute to innate immunity against microbial infection^10^, it is also involved in the pathogenesis of endotoxin shock, acquired immune deficiency syndrome and other diseases^11^. Inflammation derived from infection likely contributes to the development of BRONJ^12,13^. There are still other potential mechanisms associated with Caspase 1, IL-1β and BRONJ that need to be investigated^14^.

Sufficient evidence suggests that epigenetic alterations mediate the development of inflammation by regulating inflammatory gene transcription^15^. Histone amino-terminal tails are subject to multiple post-translational modifications, such as phosphorylation, ubiquitination, acetylation and methylation. These modifications determine chromatin structure and transcriptional outcomes^16^. Histone methylation is related to both repression and activation of transcription. Tri-methylated Lys 27 on histone H3 (H3k27me3) is considered to be critical for epigenetic repression in controlling chromatin organisation and gene silencing, and this modification is catalysed by the histone methyltransferase enhancer of zeste homologue 2 (Ezh2)^16^. Two related JmjC-domain-containing proteins, lysine demethylase 6 A (Kdm6a) and Kdm6b, can specifically mediate demethylation of H3k27me3/2 peptides, which is associated with epigenetic activation. Kdm6a and Kdm6b catalytic activities are critical determinants of proinflammatory gene expression in human primary macrophages^17^. A small molecular inhibitor, GSK-J4, was developed as a pharmacological tool to inhibit both Kdm6 members^17^. Whether the underlying pathophysiology of BRONJ is regulated by Kdm6a and Kdm6b remains to be determined.

In this study, we established in vitro and in vivo BRONJ models and examined Caspase 1, and IL-1β as direct targets of Kdm6a and Kdm6b by mediating the methylation status of H3k27 on at the promoter in macrophages. We also detected the potential anti-BRONJ role of Kdm6 inhibitors. Our work will help understand the pathologic role of Kdm6a and Kdm6b in Zol-promoted inflammatory responses and further support Kdm6a and Kdm6b as therapeutic targets for the treatment of BRONJ.

## Results

### Kdm6a and Kdm6b are involved in Zol-induced Caspase 1 and IL-1β expression in RAW264.7 macrophages

**Fig. 1:**
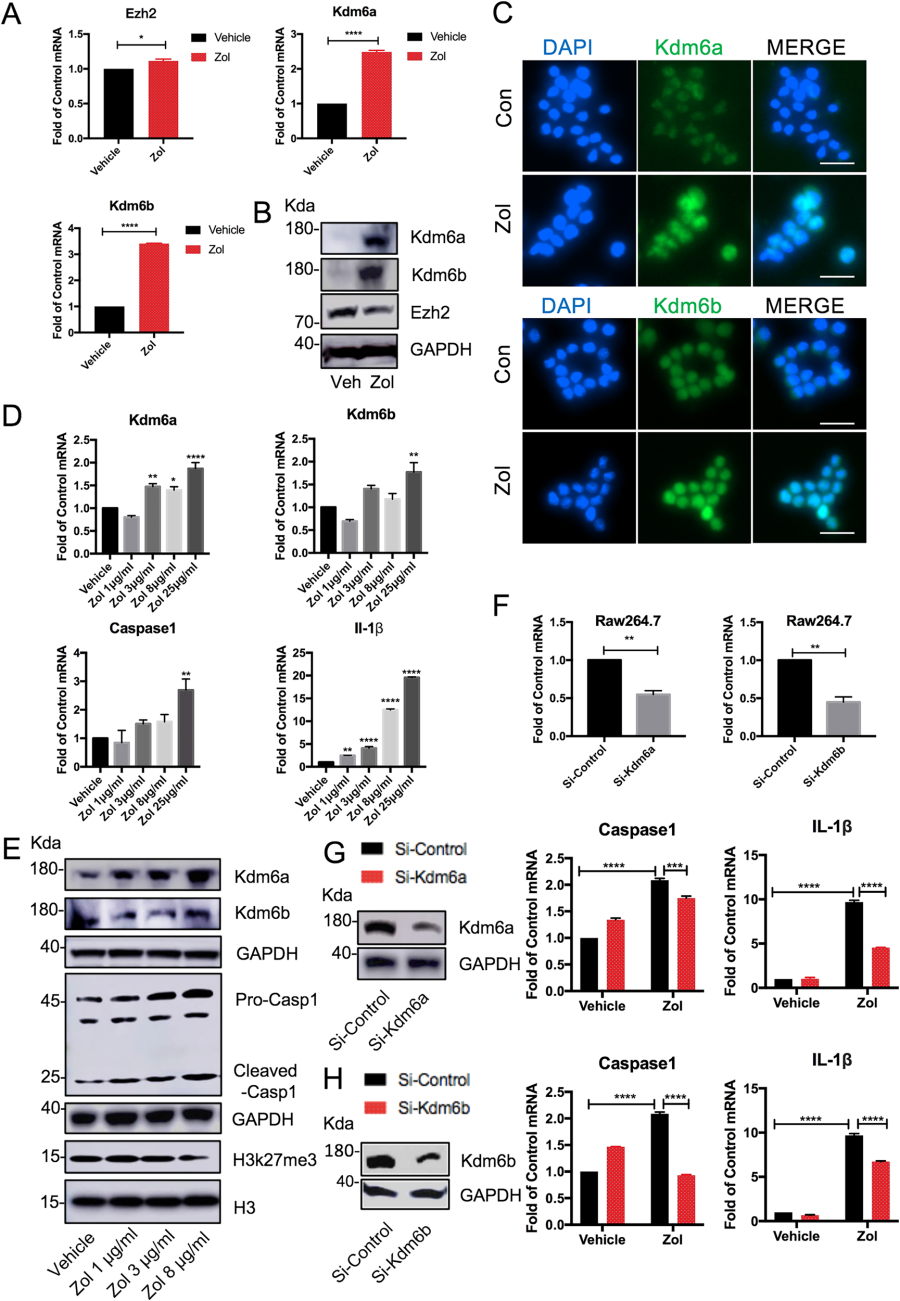
Zol treatment induces Kdm6a and Kdm6b upregulation in RAW264.7 macrophages. **a** After the Raw264.7 macrophages were incubated with 25 μg/ml Zol for 24 h, the mRNA levels of Kdm6a, Kdm6b and Ezh2 were quantified using quantitative real time-PCR. All data are analysed with Unpaired *t*-test and presented as the means with SEM, *n* = 3, *p < 0.05, ****p < 0.0001. **b** Immunoblots of Kdm6a, Kdm6b and Ezh2 in RAW264.7 macrophages treated with Zol for 48 h. **c** Representative immunofluorescent assays of RAW264.7 macrophages exposed to 25 μg/ml Zol for 48 h. Kdm6a or Kdm6b (green) and nuclei (blue) were labelled in the cells. (Scar bar = 250 μM). **d** RAW264.7 macrophages were incubated with the indicated doses of Zol for 24 h. The mRNA levels of Kdm6a, Kdm6b, Caspase 1 and IL-1β were quantified using quantitative real time-PCR. All data are analysed with Ordinary one-way ANOVA and presented as the means with SEM, *n* = 3, *p < 0.05, **p < 0.01, ****p < 0.0001. **e** Immunoblots of Kdm6a, Kdm6b, Caspase 1 and H3k27me3 in RAW264.7 macrophages treated with various doses of Zol for 48 h. **f–h** The efficiency of knockdown was confirmed by qPCR and western blotting 24 h or 48 h after transfection of Si-Kdm6a or Si-Kdm6b, respectively. The qPCR data are analysed with Unpaired t-test. The mRNA expression levels of Caspase 1 and IL-1β were assessed after transfection for 24 h and Zol-stimulated macrophages for another 24 h. The qPCR data are analysed with two-way ANOVA. All data are presented as the means with SEM, *n* = 3. **p < 0.01, ***p < 0.001, ****p < 0.0001.

To investigate whether histone methylation plays a role in the Zol-induced inflammatory responses, RAW264.7 macrophages were stimulated with vehicle or 25 μg/ml Zol. Kdm6a and Kdm6b were significantly increased at both the mRNA and protein level by the Zol incubation compared with vehicle-treated RAW264.7 macrophages (Fig. 1a, b). Although there was a slight increase in Ezh2 mRNA expression in Zol-treated cells, Zol did not consistently increase the protein level of Ezh2 (Fig. 1a, b). Expression of both Kdm6a and Kdm6b was obviously increased in the Zol-treated macrophages (Fig. 1a–c). Zol significantly increased the mRNA and protein levels of Kdm6a and Kdm6b in a dose dependent manner (Fig. 1d, e). Furthermore, Zol incubation stimulated mRNA expression of Caspase 1 and IL-1β (Fig. 1d). Zol treatment decreased H3k27me3, and elevated increased pro- and cleaved Caspase 1 (Fig. 1e). To determine whether manipulation of Kdm6a and Kdm6b affected the Zol-induced expression of Caspase 1 and IL-1β, Kdm6a and Kdm6b were knocked down (Fig. 1f–h). Knockdown of Kdm6a and Kdm6b with specific siRNAs suppressed the expression of Caspase 1 and IL-1β in Zol-stimulated RAW264.7 macrophages (Fig. 1g, h). These findings suggest that the upregulation of Kdm6a and Kdm6b in macrophages is indispensable for Zol-enhanced Caspase 1 and IL-1β activation.

### Kdm6a and Kdm6b regulate H3k27me3 at the Caspase 1 and IL-1β gene promoters in Zol-treated cells

**Fig. 2:**
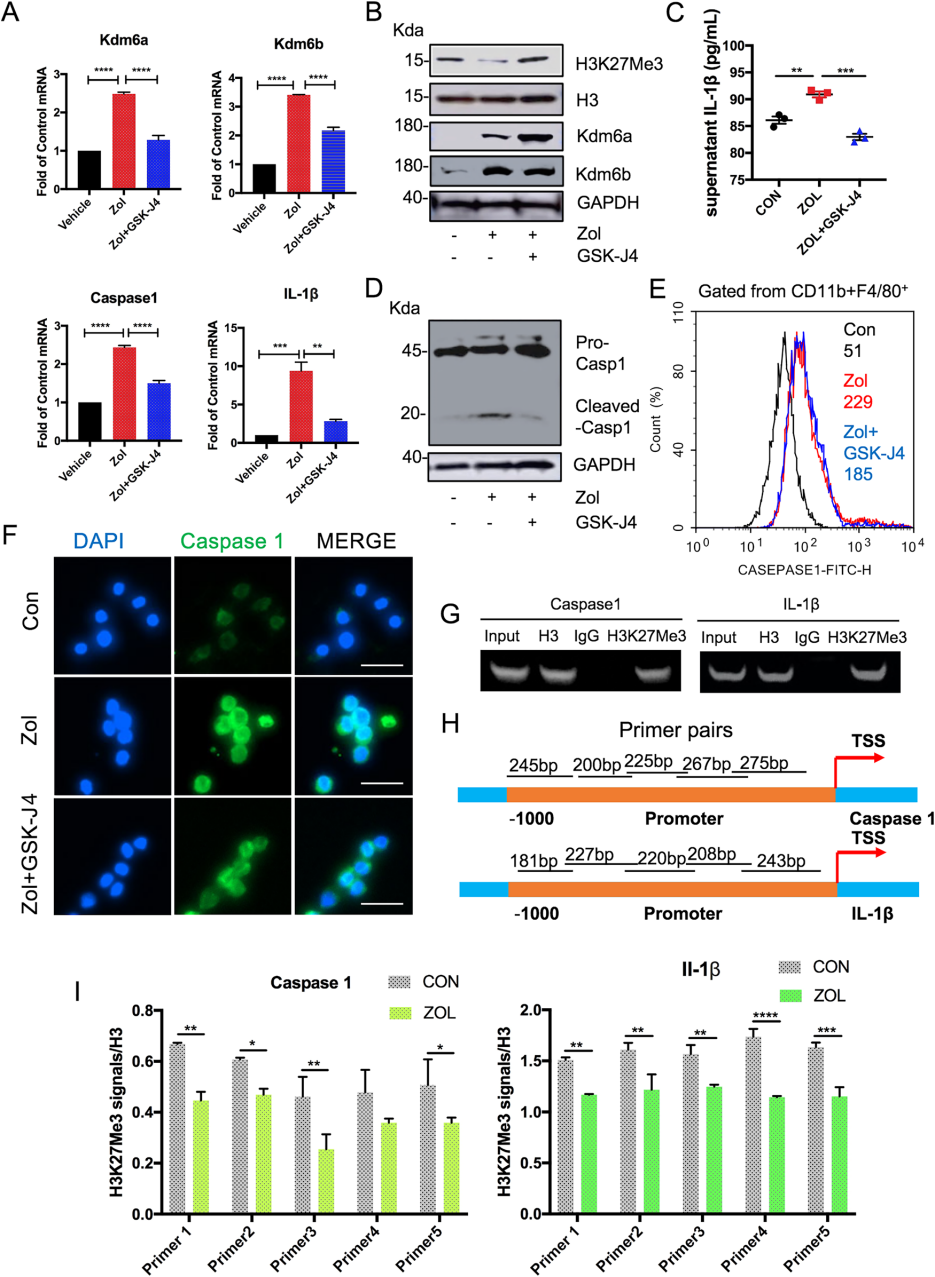
Kdm6a and Kdm6b regulate H3k27me3 at the Caspase 1 and IL-1β gene promoters and are involved in Zol-induced Caspase 1 and IL-1β expression. **a** Raw264.7 macrophages were incubated with 25 μg/ml Zol for 24 h with or without 1 μM GSK-J4 treatment, and mRNA levels of several genes were quantified using quantitative real time-PCR. All data are analysed with Ordinary one-way ANOVA and presented as the means with SEM, *n* = 3, **p < 0.01, ***p < 0.001, ****p < 0.0001. **b** RAW264.7 macrophages were exposed to 25 μg/ml Zol for 48 h with or without 1 μM GSK-J4 treatment. The cell lysates were immunoblotted with Kdm6a, Kdm6b and H3k27me3 antibodies. **c** IL-1β in the supernatant of Raw264.7 macrophages after treatment with 25 μg/ml Zol for 48 h with or without 1 μM GSK-J4 were analysed by ELISA. All data are analysed with Ordinary one-way ANOVA and presented as means with SEM, *n* = 3, **p < 0.01, ***p < 0.001. **d** RAW264.7 macrophages were exposed to 25 μg/ml Zol for 48 h with or without 1 μM GSK-J4 treatment. The cell lysates were immunoblotted with Caspase-1 antibodies. **e** Caspase 1 activity was observed by flow cytometry after treatment with 25 μg/ml Zol for 48 h with or without 1 μM GSK-J4. Values indicated in the histograms represented the mean fluorescence intensity of cells. **f** Caspase 1 expression was analysed by Immunofluorescence after treatment with 25 μg/ml Zol for 48 h with or without 1 μM GSK-J4. **g** PCR amplification was carried out using specific Caspase-1 and IL-1β upstream primers from input DNA and immuoprecipitated DNA was pulled down by H3 antibody, rabbit IgG secondary antibody and H3k27me3 antibody. **h** The region covered by the primer sets used for ChIP are shown. **i** ChIP results are expressed as the H3k27me3 signals/H3 at the Caspase 1 and IL-1β gene promoters. All data are are analysed with two-way ANOVA and presented as the means with SEM, *n* = 3. *p < 0.05, **p < 0.01, ***p < 0.001, ****p < 0.0001.

To examine whether Caspase 1 and IL-1β were regulated epigenetically, the Kdm6 family Inhibitor GSK-J4 was used. As indicated in Fig. 2a, GSK-J4 treatment significantly decreased the Zol-mediated mRNA expression of Caspase 1 and IL-1β. H3k27me3 levels in RAW264.7 cells were upregulated after GSK-J4 incubation (Fig. 2b). The level of IL-1β in Zol-treated cells was significantly higher compared with those of the control groups (Fig. 2c). Treatment with GSK-J4 significantly decreased the secretion of IL-1β (Fig. 2c). Although there was a slight decrease in Caspase 1 expression and fluorescence intensity in cells co-treated with Zol and GSK-J4 (Fig. 2e, f), cleaved Caspase 1, the active form of Caspase 1, was dramatically decreased in the GSK-J4 treated group (Fig. 2d).

To elucidate whether Zol-upregulated Caspase 1 and IL-1β resulted from decreased H3k27me3 modification at the promoter of target genes, ChIP-PCR analysis was performed on RAW264.7 macrophages. As indicated in Fig. 2g, the ChIP-PCR examination detected direct H3k27me3 modification on Caspase 1 and IL-1β promoters. There was a reduction in H3k27me3 occupation at the five regions of the promoters that were examined in Zol-stimulated RAW264.7 macrophages (Fig. 2h, i).

Taken together, these findings suggest prominent roles of Kdm6a and Kdm6b in Zol-induced inflammatory responses. The increased Caspase 1 and IL-1β expression in Zol-stimulated RAW264.7 macrophages resulted from epigenetic reduction in the repressive marker H3k27me3.

### Kdm6a and Kdm6b are involved in Zol-mediated canonical Caspase-1-dependent pyroptosis in RAW264.7 macrophages

**Fig. 3:**
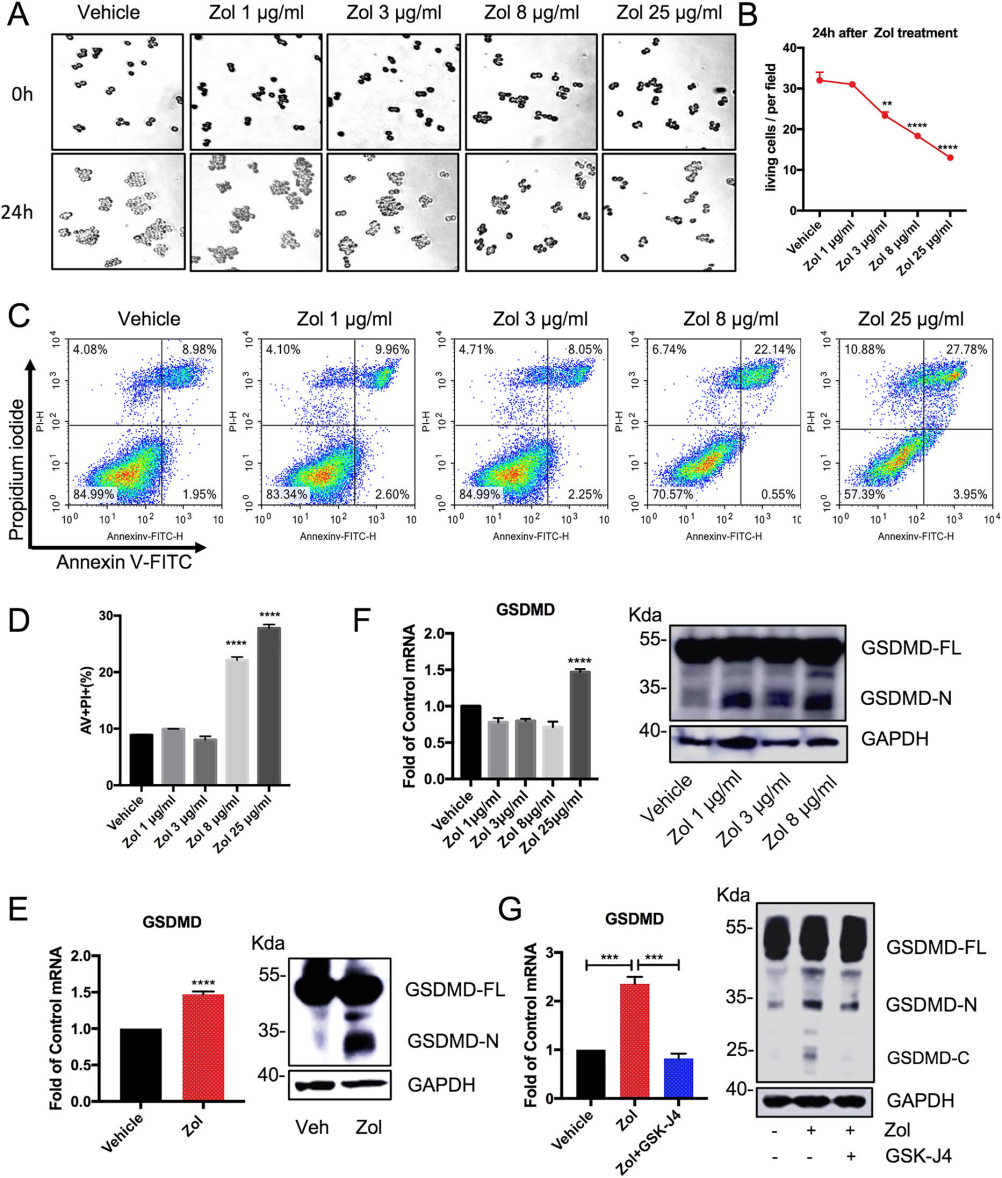
Zol induces pyroptosis via GSDMD in RAW264.7 macrophages and GSK-J4 decreases the activation of GSDMD. **a, b** Representative images of RAW264.7 macrophages incubated with the indicated doses of Zol for 24 h. The cells were counted in three randomly selected fields (50 × 50 μm per field) at the same magnification and averaged. Data are analysed with Ordinary one-way ANOVA and presented as the mean ± SEM, *n* = 6–8. **p < 0.01, ****p < 0.0001. **c, d** The cell death of RAW264.7 macrophages was measured in flow cytometer after treatment with indicated doses of Zol for 48 h. Data are analysed with Ordinary one-way ANOVA and presented as the mean ± SEM, *n* = 3. ****p < 0.0001. **e, f** The mRNA and protein levels of GSDMD were quantified using quantitative real time-PCR or western blotting, respectively. The qPCR data are analysed with Unpaired t-test in Fig. E and with Ordinary one-way ANOVA in Fig. F. All data are presented as the means with SEM, *n* = 3. ****p < 0.0001. **g** The mRNA levels of GSDMD were quantified using quantitative real time-PCR after incubation with 25 μg/ml Zol for 24 h with or without 1 μM GSK-J4. The cell lysates were immunoblotted with GSDMD antibodies. All data are analysed with Ordinary one-way ANOVA and presented as the means with SEM, *n* = 3. ***p < 0.001.

To examine the influence of Zol on cell viability, the cell numbers were counted in groups of cells treated with different doses of Zol. The cell viability was significantly inhibited by Zol incubation in a dose dependent manner (Fig. 3a, b). Cell death was induced by Zol stimulation, as measured by double-positive staining of annexin V and propidium iodide (Fig. 3c, d). The pyroptosis key executor GSDMD was upregulated at the mRNA level, and efficient cleavage of GSDMD and the N-terminal fragment were observed after Zol stimulation (Fig. 3e, f). To further investigate whether Kdm6a and Kdm6b are involved in the canonical Caspase-1-dependent pyroptosis via GSDMD, the inhibitor GSK-J4 was combined with Zol treatment. Intriguingly, GSK-J4 obviously blocked the Zol-induced transcription of GSDMD mRNA to a level equivalent to that of the control group. GSDMD was not efficiently cleaved when the Raw264.7 cells were co-stimulated with Zol and GSK-J4 compared to that of cells treated with only Zol (Fig. 3g). This can explain the decreased release of IL-1β by Zol-treated cells following treatment with GSK-J4 (Fig. 2c). These data indicated that Kdm6a and Kdm6b play important roles in IL-1β secretion in Zol-treated macrophages through the canonical Caspase-1-dependent pyroptosis pathway.

### Blocking Kdm6a and Kdm6b activation rescues poor healing of alveolar socket in Zol-treated mice

**Fig. 4:**
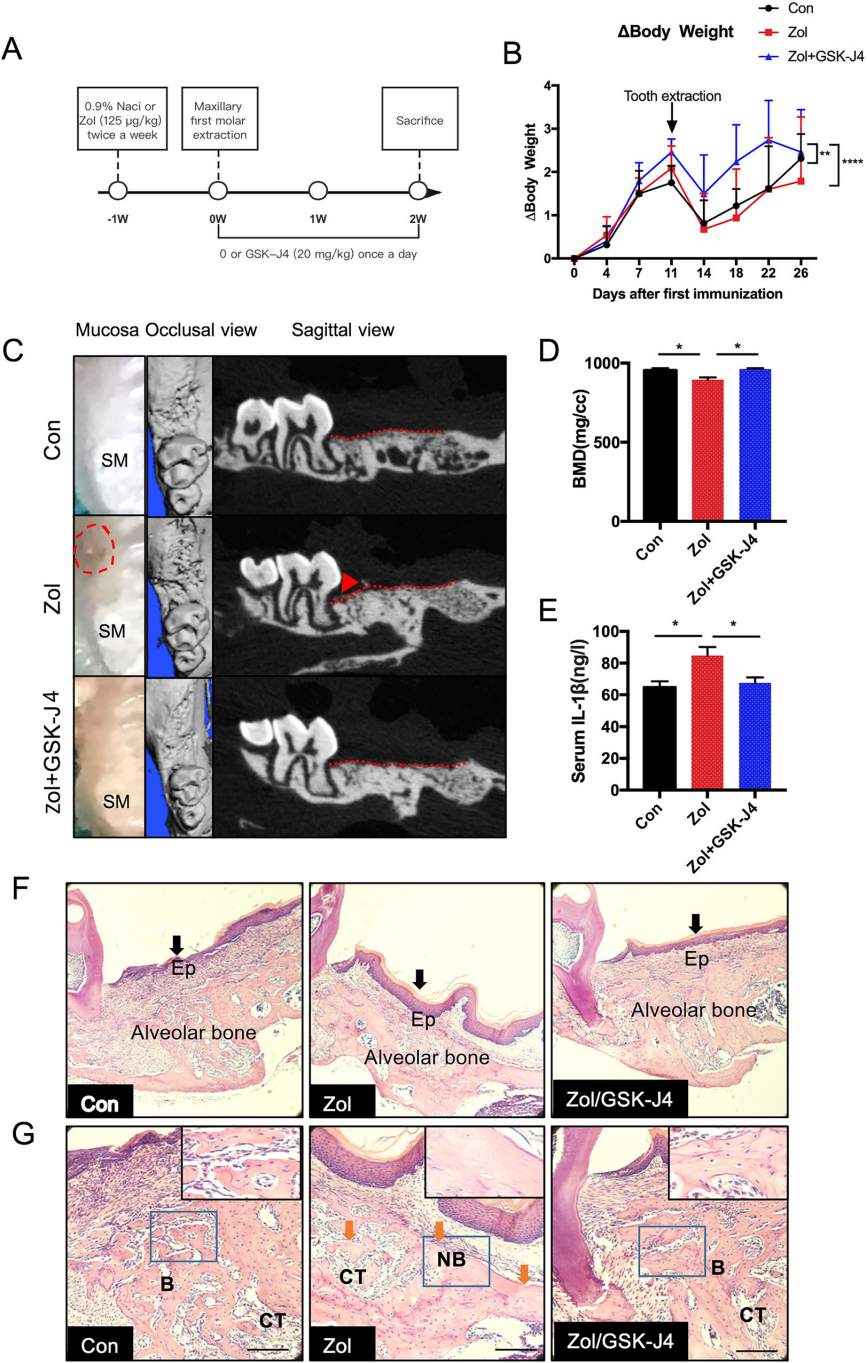
Blocking Kdm6a and Kdm6b activation improved the healing of alveolar socket in Zol-treated mice. **a** A graphical representation of the experimental protocol used in this study. **b** The body weight of mice was monitored twice a week. Data are analysed with two-way ANOVA (compare column means) and presented as the mean ± SEM, *n* = 6–8. **p < 0.01, ****p < 0.0001. **c**. Illustrations of gingival mucosa at the extraction sites at the end of 2 weeks. The red dashed circle represents an image of the delayed healing of gingival mucosa at the extracted site adjacent to the second molar (SM). Micro-CT analysis taken from the occlusal and sagittal views. Red lines showed the level of the bone. Red triangles showed reduced bone formation and wide periodontal ligament (PDL) space near SM in Zol-treated mice but not in mice treated with Zol plus GSK-J4. **d** The bone mineral density (BMD) were analysed. Data are analysed with Ordinary one-way ANOVA and presented as mean ± SEM, *n* = 3. *p < 0.05. **e** The serum levels of IL-1β were determined by ELISA. Data are analysed with Ordinary one-way ANOVA and presented as the mean ± SEM, *n* = 4–6. *p < 0.05. **f** Histological images of the extraction sockets showing healed gingival mucosa with epithelial coverage (black arrow) (10×). **g**. H&E staining of extraction socket areas displaying newly formed bone (B), connective tissues (CT) and necrotic bone (NB) (orange arrow) (20×). (Scale bars = 250 μm).

Next, we established an in vivo model of BRONJ to further examine whether blocking Kdm6a and Kdm6b impedes the development of BRONJ-like lesions. The mouse model in this study is described in Fig. 4a. The body weight of the mice was recorded twice a week. Overall, Zol treatment had little effect on body weight compared to that of the control group (*p* > 0.05). However, the body weight was increased more in the Zol+GSK-J4 group compared to those of the control (*p* < 0.01) and Zol groups (*p* < 0.0001) (Fig. 4b).

The clinical examination at the end of second week post tooth extraction revealed that none of the mice had an open alveolar socket without mucosal coverage or exposed necrotic bone. Only one mouse in the Zol group had delayed healing of the oral mucosa, which is consistent with the low incidence of BRONJ according to an epidemiologic study^18^. Radiographic evaluation by microcomputed tomography (microCT) showed the presence of a poorly defined alveolar ridge owing to impaired bone healing, mottled trabecula in the alveolar sockets and wide periodontal ligament (PDL) space near the second molar (SM) in mice treated with Zol only. Notably, treatment with GSK-J4 markedly promoted mucosal healing and woven bone formation in Zol-treated mice. It also markedly rescued poor healing of the alveolar socket and attenuated disease severity in Zol-treated mice (Fig. 4c). A significant decrease in the bone mean density (mg/cc) of extraction sockets was observed in Zol-treated mice compared with those of their control and rescue group littermates (Fig. 4d). In addition, we analysed the levels of IL-1β in mouse serum at second week post tooth extraction. Zol treatment increased the serum levels of IL-1β, whereas treatment with GSK-J4 decreased the serum levels of IL-1β (Fig. 4e).

Histological examination showed that the tooth extraction sockets in control mice underwent a regular course of healing with epithelial migration, bone regeneration and complete oral mucosal closures above the alveolar socket (black arrow) within 2 weeks (Fig. 4f). The phenotype of the Zol only groups showed complete oral mucosal closures without epithelial migration and bone regeneration. Further analysis showed that necrotic bone (orange arrow) characterised by diffuse loss of osteocytes with confluent areas of empty lacunae (G inset) in Zol-treated mice. These effects were significantly rescued in the presence of GSK-J4 (Fig. 4g), indicating that blocking Kdm6a and Kdm6b ameliorated poor healing of alveolar socket in mice.

**Fig. 5:**
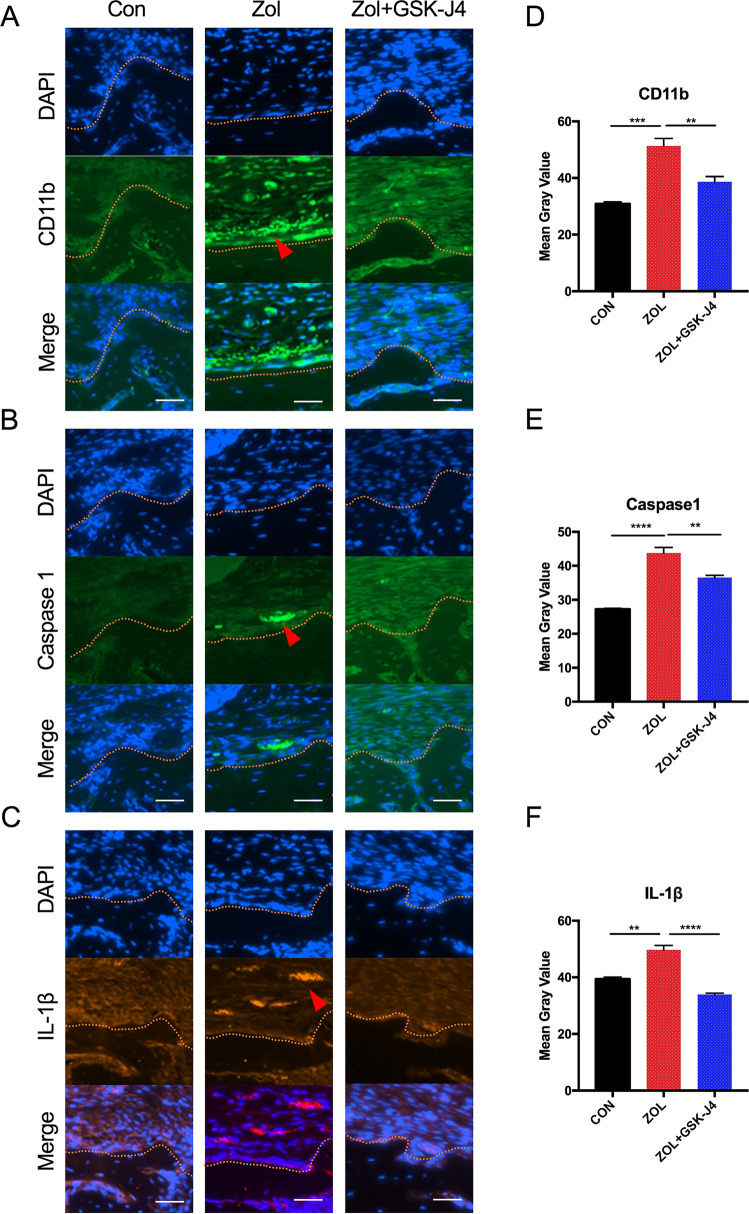
Blocking Kdm6a and Kdm6b activation suppressed inflammatory cytokines expression in Zol-treated mice. **a–c** Paraffin-embedded sections of tooth extraction sites of mice in all groups were immunostained for CD11b, Caspase-1 and IL-1β. Nuclei were stained with DAPI. Red triangles showed positive cells almost gathering in the soft tissue wounds near bone surface. (Scale bars = 40 μm). Yellow dotted lines indicate the border between alveolar bone and connective tissues. **d–f** Mean grey values of CD11b, Caspase 1 and IL-1β were quantified. All data are analysed with Ordinary one-way ANOVA and presented as the means with SEM, *n* = 3. **p < 0.01, ***p < 0.001, ****p < 0.0001.

Immunofluorescence studies showed that the local socket wound sites of Zol-treated mice constitutively expressed higher protein levels of Caspase-1 and IL-1β than those of mice in other groups. CD11b^+^ macrophages were also detected in soft tissue wounds near bone surface in Zol-treated mice. Moreover, most of the necrotic bone was found adjacent to the area of intense local inflammation with high expression of Caspase-1 and IL-1β, suggesting an association between inflammation and tissue degeneration in the extraction sockets of Zol-treated mice. In the GSK-J4 rescue group, the expression of CD11b, Caspase-1 and IL-1β are lower than that of the Zol-treated group (Fig. 5a–c). Mean grey values of CD11b, Caspase 1 and IL-1β were quantified (Fig. 5d–f). These results suggest a potential role for GSK-J4 in preventing BRONJ-like lesions in mice.

## Discussion

Macrophages are key regulators of innate immunity during microbial infection and are an essential component of chronic inflammatory diseases and also in fighting infectious agents^19^. Inflammation is a tightly regulated response of the innate immune system to tissue damage and pathogen invasion and aims to restore tissue homoeostasis^20^. Various inflammatory interleukins such as IL-1β have crucial roles in this process. The cytokine is synthesised as inactive precursors and is cleaved by active Caspase-1 to generate the mature active forms^21^. Proinflammatory Caspase-1 also activates GSDMD, an important executor of a type of programmed cell death called pyroptosis. GSDMD is inextricably related to Caspase 1 activation both as a downstream effector and as a participant of IL-1β secretion^9^. A growing body of evidence has shown that macrophage function may potentially be altered by BPs and contributes to defective healing in BRONJ^22,23^. Caspase-1 and IL-1β activation have a tight regulation on BRONJ and other autoinflammatory diseases^24,25^. Admittedly in our study, we used the Raw264.7 cells instead of bone marrow-derived macrophages. We found high expression of Caspase 1 and IL-1β in Zol-treated RAW264.7 cells. We also found single Zol treatment slightly, but significantly, stimulated IL-1β secretion as previously reported^4^. But Zol can enlarge the effect of the other stimulus in the microenvironment of BRONJ, such as LPS. In this study, we choose single Zol treatment to macrophages, because we aim to pinpoint the mechanism of zol induced IL-1β secretion and exclude the interference of other factors. The N-terminus of GSDMD, which is important for IL-1β secretion, was detected with Zol stimulation. We can find that low dosage of Zol induced GSDMD cleavage but did not increase cell death. In fact, GSDMD executes inflammatory Caspases-induced pyroptosis by generating pores on the plasma membrane. GSDMD cleavage and the formation of GSDMD pores can also mediate IL-1β secretion without causing cell death. Macrophages can utilise inflammasome-dependent processes to activate GSDMD pore formation and remain viable which is called cell hyperactivation. Also, hyperactive cells (living cells) display evidence of lower amount of GSDMD pores than pyroptotic cells (dead cells). However, the mechanisms that permit GSDMD to either promote pyroptosis or cell hyperactivation are unclear^26,27^. So we suppose that Zol in low dose can stimulate macrophages to generate GSDMD pores and cause cells in hyperactive state. However, this needs further investigation. Mice treated with Zol alone had impaired alveolar ridges and necrotic bone in the wounds, which is consistent with previous studies^28^. It is reported that osteal macrophages, typically classified as being pro- or anti-inflammatory in nature, are vital regulators of bone. And the inflammatory state can result in decreased bone mass^29^. The osteal macrophages usually can be seen spread in the plane of the bone surface^30^. We visualised CD11b-positive macrophages and found high expression of Caspase 1 and IL-1β in extraction wounds near bone surface of Zol-treated mice. So we consider that targeting of the inflammatory responses of macrophages and Caspase 1 and IL-1β expression highlights a potential therapeutic strategy for BRONJ treatment.

Histone lysine demethylases Kdm6a and Kdma6b are chromatin modifiers that play important roles in many pathological processes, such as inflammation and cancer, making them potentially attractive drug targets^31^. Thy are also reported to be crucial for eukaryotic transcription, genome integrity and epigenetic inheritance^32–34^, as well as in development and physiology diseases^35^. Kdm6a exhibits high homology and structural similarity to Kdm6b, especially in the JmjC domain. They can specifically demethylate tri-methylated Lys 27 on histone H3, which in general is associated with transcriptional repression and regulating a number of cellular processes such as development, differentiation and proliferation^36^. However, very few studies have focused on the role of demethylases Kdm6a and Kdm6b in the activation of Caspase 1, which is associated with the inflammatory responses induced by NBPs and the development of BRONJ^4^. In this study, Kdm6a and Kdm6b were associated with Caspase 1 activation and GSDMD cleavage. Increased Kdm6a and Kdm6b were found in Zol-treated RAW264.7 cells. Moreover, Kdm6a and Kdm6b depletion or inactivation decreased the Zol-induced activation of Caspase 1, and synthesis and secretion of IL-1β. Depletion of Kdm6a and Kdm6b also decreased GSDMD cleavage, which is consistent with the reduced secretion of IL-1β. The in vivo experiment results showed that GSK-J4 rescued the atypical healing of alveolar sockets in Zol-treated mice and suppressed CD11b-positive macrophages and the expression of Caspase1 and IL-1β in extraction wounds near bone surface, further supporting that Kdm6a and Kdm6b were involved in the regulation of inflammation and the pathogenesis of BRONJ.

To understand the underlying mechanisms, we performed ChIP-PCR assay to investigate the trimethylation status of histone H3k27 sites on the Caspase 1 and IL-1β promoters in macrophages. We identified Caspase 1 and IL-1β as direct targets of Kdm6a and Kdm6b by mediating the methylation status of H3k27 on the promoters and Zol decreased the repressive marker H3k27me3 on the Caspase 1 and IL-1β promoters. Thus, we hypothesised that the decreased trimethylation of histone H3k27 was likely involved in the abnormal transcription of Caspase 1 and IL-1β. Together, these data demonstrated that Kdm6a and Kdm6b promote Caspase 1 and IL-1β expression by directly binding to the promoter regions of the Caspase 1 and IL-1β genes, reducing H3k27me3 occupancy, and increasing IL-1β synthesis and GSDMD cleavage, which in turn caused pyroptosis and secretion of IL-1β.

**Fig. 6:**
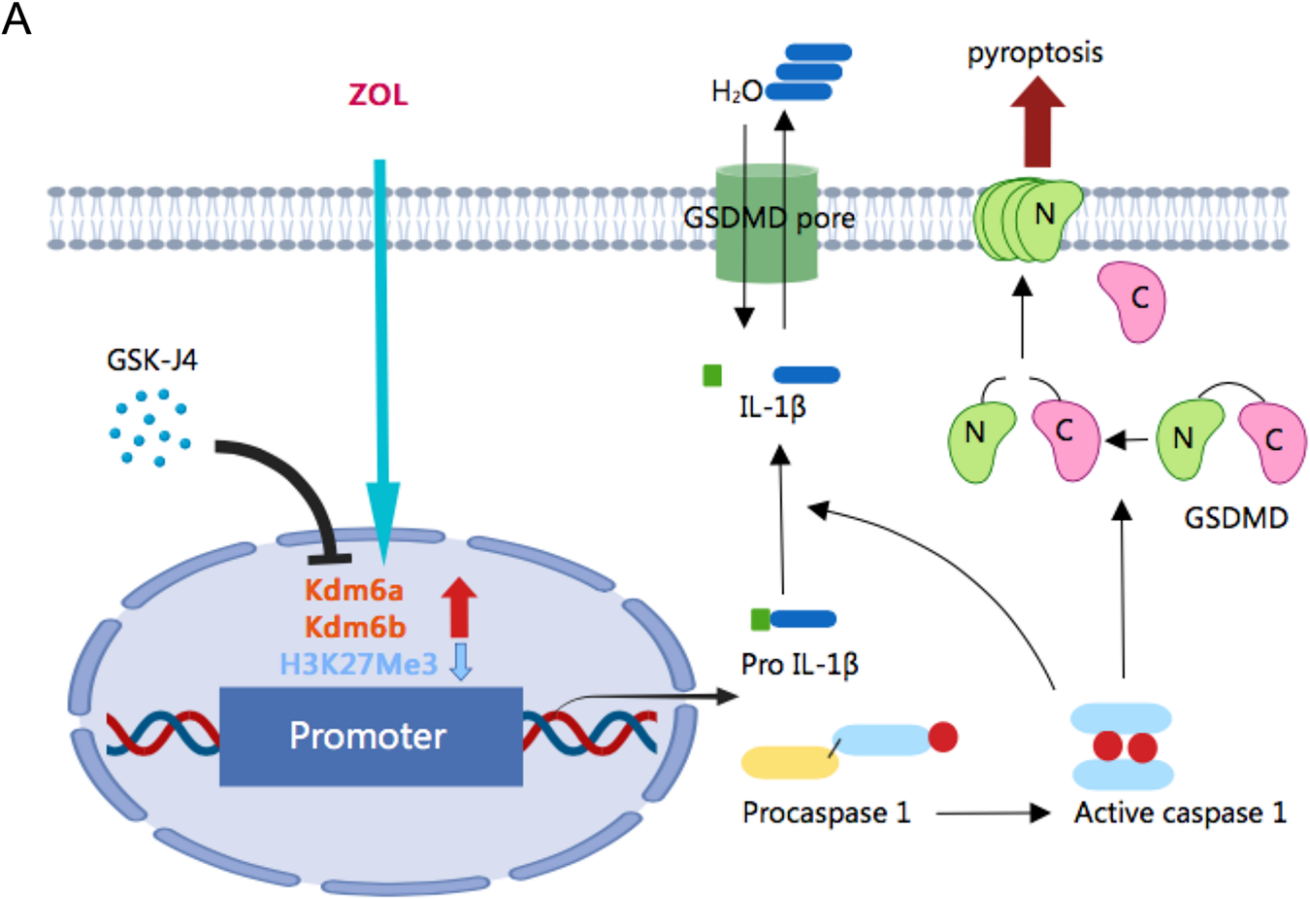
The proposed model for histone demethylase Kdm6a and Kdm6b regulation of Zol-mediated inflammatory responses. Zol increased the expression of Kdm6a and Kdm6b. The increased Kdm6a and Kdm6b led to reduced H3k27me3 modification at the promoters of the Caspase 1 and IL-1β genes, which resulted in the elevated transcription of Caspase 1 and IL-1β and GSDMD mediated IL-1 β secretion.

In summary, our findings support the hypothesis that the upregulation of Kdm6a and Kdm6b in macrophages plays an important role in Caspase 1 activation and IL-1β synthesis and secretion (Fig. 6). These findings extend the knowledge of epigenetic regulation in inflammation and further elucidate the pathogenesis of BRONJ.

## Methods and materials

### Reagent sources

The murine macrophage-like RAW 264.7 cell line was obtained from the American Type Culture Collection (Maryland, USA); Antibodies against Kdm6a, Kdm6b, H3, H3k27me3 and GAPDH were purchased from Cell Signalling Technology (Beverly, MA, USA); GSDMD was obtained from Abcam (London, UK); Caspase 1 antibody and goat anti-rabbit IgG (H + L) FITC were generated by ABclonal Biotechnology (Wuhan, China); The Kdm6a and Kdm6b inhibitor GSK-J4 was purchased from Selleck Chemicals LLC (Houston, TX, USA); APC anti-mouse CD11b antibody and PE anti-mouse F4/80 antibody were purchased from BD Biosciences (New Jersey, USA); Foetal bovine serum was purchased from Life Technologies (now Thermo Fisher Scientific Inc, NY, USA); Zol was generated by Novartis Pharma Schweiz AG (Basel, Switzerland).

### Cell culture and treatment

RAW264.7 cells were cultured in complete DMEM (HyClone) supplemented with 10% foetal bovine serum (FBS) and 1% penicillin/streptomycin in a humidified atmosphere with 5% CO_2_ at 37 °C. The cells were stimulated with 25 μg/ml Zol with or without a specific inhibitor.

### Animal care

All experimental protocols using animals conformed to the Animal Welfare Act Guide for the Use and Care of Laboratory Animals and were approved by the Institutional Animal Care and Use Committee of Shanghai Jiao Tong University in China. Eight-week-old female C57BL/6 mice were purchased from Jackson Laboratory. All mice were kept in a pathogen-free vivarium in the Shanghai Key Laboratory of Stomatology & Shanghai Research Institute of Stomatology according to the approved institutional guidelines of Shanghai Jiao Tong University. The mouse model for BRONJ used in this study is described below. Briefly, mice were divided into three groups randomly (*n* = 6–8 per group) and received administration of Zol (125 µg/kg) or vehicle solution (0.9% NaCl saline) intraperitoneally twice a week throughout the experiment. One week after ip administration, the maxillary first molars were extracted under chloral hydrate anaesthesia and allowed to heal. For BRONJ-rescue experiments, GSK-J4 (20 mg/kg) was administered daily after the maxillary first molars were extracted, and the mice were allowed to heal for another 2 weeks. The body weight of the animals was recorded biweekly. Finally, the maxillae were harvested for further analysis by other investigators who were blinded to the group allocation. The mice with remaining roots in the extraction sockets were excluded from subsequent analysis during the next radiographic evaluation.

### Micro-computed tomography (microCT) scanning and three-dimensional volumetric analysis

The harvested maxillae were fixed with 4% paraformaldehyde at 4°C overnight and stored in a 70% ethanol solution. The fixed maxillae were scanned by a microcomputed tomography scanner (microCT35; Scanco Medical AG, Bassersdorf, Switzerland) with a 14 µm voxel size. Two-dimensional slices from each maxilla were combined using microCT v6.1 software (Scanco Medical) to form a three-dimensional reconstruction. Reconstructions were further analysed as previously described^18^. The region of interest (ROI) was defined to cover the whole tooth extraction socket. The bone mean density (BMD) of each extraction site was measured semi-automatically in accordance with the guidelines for the assessment of bone microstructures using microCT^37^.

### Histological assessments

After microCT scanning, these tissues were decalcified with 5% EDTA and 4% sucrose in PBS (pH 7.4). Decalcification continued for 1 month at 4 °C. The decalcification solution was changed daily. Tissue samples were processed for paraffin embedding. Briefly, 5 mm thick samples were stained with haematoxylin and eosin (H&E). Necrotic bone in the extraction sockets was defined as the portion of bone in which there were greater than or equal to 10 adjacent empty or pyknotic osteocyte lacunae because such bone is not alive^28^. For dual-colour immunofluorescence studies, sections were immunostained with specific antibodies for the murine macrophage markers CD11b, Caspase-1 and IL-1β, followed by staining with FITC- and rhodamine-conjugated secondary antibodies (1:400). Nuclei were stained with DAPI. Mean grey values of CD11b, Caspase 1 and IL-1β were quantified with ImageJ software (three fields were randomly selected at the same magnification).

### Western blot analysis

An equal amount of protein from each cell lysate was resolved on an SDS-polyacrylamide gel and was electroblotted onto a polyvinyl difluoride membrane (Millipore, USA). After blocking with 5% nonfat dry milk to avoid the interference of nonspecific binding for 1 h, the membrane was incubated overnight at 4 °C with primary antibody, followed by incubation with an anti-rabbit HRP-conjugated secondary antibody (1:5000, Cell Signalling Technology) for 1 h. Immunoreactive proteins were visualised by enhanced chemiluminescence (ECL) detection (Thermo Scientific, Waltham, MA, USA). GAPDH was used as an internal control.

### Real-time quantitative RT-PCR analysis

Total RNA extracted from RAW264.7 cells was isolated with the Trizol Reagent (Invitrogen, Carlsbad, CA, USA). Total RNA was reverse-transcribed into complementary DNA (cDNA) using a two-step RT kit (Takara Biotechnology, Dalian, China) according to the manufacturer’s instructions. Actin was used as an internal standard for mRNA analysis. Relative gene expression was determined by 2^−∆∆Ct^. See Table 1 for the primer sequences.

**Table 1:** Primers used for qRT-PCR validation.

Gene name	Primer name	Primer sequence (5’ to 3’)
Actin	Mouse Actin forward	CATGTACGTTGCTATCCAGGC
	Mouse Actin reverse	CTCCTTAATGTCACGCACGAT
Kdm6a	Mouse Kdm6a forward	AAGGCTGTTCGCTGCTACG
	Mouse Kdm6a reverse	GGATCGACATAAAGCACCTCC
Kdm6b	Mouse Kdm6b forward	TGAAGAACGTCAAGTCCATTGTG
	Mouse Kdm6b reverse	TCCCGCTGTACCTGACAGT
Caspase 1	Mouse Caspase 1 forward	AATACAACCACTCGTAC
	Mouse Caspase 1 reverse	AGCTCCAACCCTCGGAG
IL-1β	Mouse IL-1β forward	GCAACTGTTCCTGAACTCAACT
	Mouse IL-1β reverse	ATCTTTTGGGGTCCGTCAACT
Ezh2	Mouse Ezh2 forward	TGCCTCCTGAATGTACTCCAA
	Mouse Ezh2 reverse	AGGGATGTAGGAAGCAGTCATAC
GSDMD	Mouse GSDMD forward	CCATCGGCCTTTGAGAAAGTG
	Mouse GSDMD reverse	ACACATGAATAACGGGGTTTCC

### Immunofluorescence analysis

The cells were fixed with paraformaldehyde for 30 min, permeabilized with 0.25% Triton X-100 for 10 min, and blocked with goat serum for 30 min. The cells were incubated with rabbit anti-Kdm6a, Kdm6b or Caspase 1 antibody at 4 °C overnight, and the secondary antibodies used were FITC- conjugated goat anti-rabbit IgG (1:100 dilution). Nuclei were stained with DAPI. Pictures were taken by confocal laser scanning microscopy (Zeiss LSM780, Carl Zeiss).

### ELISA

The levels of IL-1β in the supernatants of cultured cells or in mouse serum were determined by ELISA using an IL-1β kit (Boatman Biotechnology, Shanghai, China) according to the manufacturer’s protocol. Optical densities were read on a microplate reader in this kit at 450 nm. The results are presented as picograms per millilitre.

### Apoptosis detection

Cells were collected and washed twice with cold PBS. Then, the cells were centrifuged at 5000 rpm for 5 min at 4 °C and resuspended in 1× annexin V binding buffer by using an annexin V-FITC/propidium iodide apoptosis assay kit (BD Biosciences). Next, the cells were centrifuged at 300 rcf for 10 min at 4 °C and then stained with annexin V and propidium iodide in annexin V binding buffer for 15 min on ice in the dark. Stained cells were measured in a flow cytometer (BD Biosciences, USA), and the data were processed using FlowJo software. Annexin-V has a high affinity for phosphatidylserine exposed in the external cellular environment on apoptotic cells but can also stain pyroptotic cells because of membrane rupture, and so the phosphatidylserine on the inner leaflet can also be recognised^38^.

### Small interfering RNA transfection

Mouse Kdm6a siRNA (5′-CAACAATATTGCTTGGAAT-3′), mouse Kdm6b siRNA (5′-CCAGACAGCACTACAGGAA-3′) and control siRNA (5′-GGACTACAAGAA CACTTT-3′) were produced by Sangon Biotech (Shanghai, China). To introduce the siRNA into the cells, the cells were plated into on 6-well plates at 30–50% confluence before transfection. Transfection of siRNA was carried out using riboFECT™ CP (RIBOBIO, Guangzhou, China). The efficiency of knockdown was confirmed by qPCR and western blotting. After 24 h or 48 h, transfected RAW264.7 cells were treated with Zol and analysed as indicated.

### Caspase-1 activation assay

Cells were collected after digestion and incubated with Caspase-1 primary antibody on ice for 15 min. To quantify the activity of Caspase-1 in RAW264.7 cells by flow cytometry, the cells were stained with PE anti-mouse F4/80 antibody, APC anti-mouse CD11b antibody and FITC goat anti-rabbit IgG (H + L) at 4 °C for 30 min in the dark, fixed with 4% paraformaldehyde for 20 min and washed with PBS twice. The fluorescence intensity of Caspase-1 was detected by flow cytometry (BD Biosciences, USA), and the data were processed using FlowJo software.

### Chromatin immunoprecipitation

The chromatin immunoprecipitation (ChIP) assay was performed using the SimpleChIP® Plus Enzymatic Chromatin IP Kit (Magnetic Beads) (Cell Signalling Technology) according to the manufacturer’s instructions. The cells were crosslinked with a final concentration of 1% formaldehyde for 10 min at room temperature. Then, 125 mM glycine was added to quench the unreacted formaldehyde. Chromatin was fragmented by partial digestion with Micrococcal nuclease to obtain chromatin fragments of 1 to 5 nucleosomes. Chromatin immunoprecipitations were performed using ChIP-validated antibodies and ChIP-grade protein G magnetic beads. After reversing the protein-DNA crosslinks, the DNA was purified using DNA purification spin columns. The enrichment of particular DNA sequences following immunoprecipitation was analysed by standard PCR and quantitative real-time PCR. PCR analysis was performed by using multiple sets of primers for the Caspase 1 and IL-1β gene promoters. See Table 2 for the primer sequences.

**Table 2:** Caspase 1 and IL-1β gene promoter primers used for CHIP-qPCR validation.

Gene name	Primer name	Primer sequence (5’ to 3’)
Caspase 1	Mouse Primer 1 forward	TGAAATCTTGAGTTCTCACTCCTCT
	Mouse Primer 1 reverse	GCCTTTTACTCTGTTTTTAGGTGTG
Caspase 1	Mouse Primer 2 forward	CACTGTCAGGGTTTATATAGGGC
	Mouse Primer 2 reverse	GCACCTTGAATTATGAGTTGGG
Caspase 1	Mouse Primer 3 forward	TATAAAACTATCCAGGACAACACCC
	Mouse Primer 3 reverse	GGCTTCTCATTTACAATAGCAACTC
Caspase 1	Mouse Primer 4 forward	CCCTAATAACTGCTCAGTGGAGTT
	Mouse Primer 4 reverse	TGATTAGATACCTGGCTCTTGGCT
Caspase 1	Mouse Primer 5 forward	GCCAGTTGTTGAAAACCATTTC
	Mouse Primer 5 reverse	GATGTAGTAGTTACCGTCTGCACCT
IL-1β	Mouse Primer 1 forward	TCCTTTGGGACCTTAGACCTGA
	Mouse Primer 1 reverse	GGCAACATGACCAAATAGAGACA
IL-1β	Mouse Primer 2 forward	TGTCTCTATTTGGTCATGTTGCC
	Mouse Primer 2 reverse	AAGGGAGTAAGGTTTGATCCTAAA
IL-1β	Mouse Primer 3 forward	TTTAGGATCAAACCTTACTCCCTT
	Mouse Primer 3 reverse	TCCACATCTAGCAAGGAACCC
IL-1β	Mouse Primer 4 forward	AGGGTTCCTTGCTAGATGTGG
	Mouse Primer 4 reverse	AGGATACTTGAGGTCTGGGGA
IL-1β	Mouse Primer 5 forward	CATCTCCCCAGACCTCAAGT
	Mouse Primer 5 reverse	TCTGCCTGGCTATTGACTTCTC

### Statistical analysis

All experiments were repeated a minimum of three times unless specifically indicated. Statistical significance is based on the analysis of at least triplicate samples. Experimental data are shown as means ± standard error of the mean (SEM). Statistical analyses were conducted by using Prism version 7.0 (GraphPad, USA). Unpaired *t*-test, Ordinary one-way ANOVA, and two-way ANOVA were selected to compare the differences among groups. The *p* value < 0.05 was considered statistically significant.
